# Recent Advances in Phenylketonuria: A Review

**DOI:** 10.7759/cureus.40459

**Published:** 2023-06-15

**Authors:** Andrea M Zuñiga Vinueza

**Affiliations:** 1 School of Medicine, Catholic University of Santiago of Guayaquil, Guayaquil, ECU

**Keywords:** management, diagnosis, infant, neonatal, phenylketonuria

## Abstract

This article highlights the significance of inborn errors of metabolism and focuses specifically on phenylketonuria (PKU), a well-known inheritance disorder caused by the deficiency or absence of phenylalanine hydroxylase (PAH). This review discusses associated mutations in the PAH gene and their impact on phenylalanine metabolism. A total of 40 articles were analyzed between 2019 and 2023, covering diagnostic innovations, advancements in treatment and management strategies, and the long-term implications of PKU. This study emphasizes the importance of early diagnosis and highlights the ongoing need for advancements in screening methods and treatment approaches to optimize patient outcomes in PKU patients. This review provides valuable insights for healthcare professionals involved in the care of children with PKU and contributes to the enhancement of clinical practice in this field.

## Introduction and background

Definitions and epidemiology

Inborn metabolic errors are inherited disorders that affect normal metabolism in the body. Genes that encode enzymes or proteins involved in metabolic pathways have mutations, which are the root cause of these disorders. Consequently, there are defects in the processing of nutrients, production of energy, or elimination of metabolic waste, which can lead to the accumulation of toxic substances or a deficiency of essential metabolic products [1-4].

PKU is one of the most well-known inborn metabolic disorders, with a prevalence of 1/100,000 live births in Europe and 1/4,000 in Türkiye [5].

It is characterized by the inability of the body to properly break down the amino acid phenylalanine (Phe). This is due to the deficiency or absence of PAH, which converts Phe into another amino acid called tyrosine. PKU affects PAH gene expression, leading to impaired Phe metabolism and its conversion to tyrosine. Consequently, Phe accumulates in the plasma, causing detrimental effects on various systems, including the nervous system (resulting in symptoms such as myoclonus, severe mental status changes, and seizures), skin (causing hypopigmentation), and urine compounds [6-11].

Several mutations are associated with PKU. The most common mutation is in PAH, which results in decreased or no enzymatic activity. Other less frequent mutations may affect genes related to the production of cofactors necessary for PAH function, such as tetrahydrobiopterin (BH4). These mutations can vary in severity and influence clinical presentation and response to treatment in individuals with PKU. Genetic analysis is used to identify the specific mutations present in each patient and can be useful for accurate diagnosis and individualized treatment planning [12,13].

A study involving 14 of 485,634 live births between January 1965 and December 2014 revealed that 64% of the patients were adults, 35% had cognitive disabilities, and 78% did not have consanguineous relationships [14]. In Chile, the IVS10 mutation has been identified as a cause of PKU (PAH gene) with a recurrence rate of 25%, similar to that observed in Spain [15].

Classic phenylketonuria (cPKU) is an inherited condition characterized by elevated levels of phenylalanine, an amino acid, in the bloodstream. Without treatment, phenylalanine accumulation can lead to severe health issues, including intellectual disability. Infants with cPKU typically show no symptoms initially but develop permanent cognitive impairment if left untreated. Seizures, developmental delays, behavioral problems, and psychiatric disorders are also common. Unmanaged PKU can cause a distinctive musty or mouse-like odor, as well as lighter skin and hair, and skin disorders like eczema in affected individuals [5].

A review of the latest research on PKU is essential for healthcare professionals because of its complex nature and impact on patient health. Advancements in screening, early diagnosis, and treatment can improve patient outcomes. Understanding PKU's long-term implications for patient development and quality of life is therefore crucial. This literature review provides valuable insights and recommendations to enhance clinical practice and overall management of PKU, justifying the importance of this topic.

## Review

Materials and methods

Study Design

The present report describes an updated literature review of innovations in the diagnosis and treatment of childhood PKU. This study will be based on open-access publications from the last five years in English and Spanish for humans. Specific objectives include defining methods for the diagnosis and treatment of PKU in childhood, evaluating innovations in early diagnosis, analyzing new treatment and management strategies, exploring advances in gene and targeted therapies specific to this disease, and summarizing key findings and recommendations for clinical practise. The inclusion criteria were open access to scientific publications, articles published within the last five years, human studies related to PKU in childhood, and publications in English and Spanish. Articles not related to the diagnosis and treatment of PKU, animal studies, and cellular models; articles in languages other than English and Spanish; and publications that were not available in open access were excluded.

This study focused on PKU as a rare inherited metabolic disease affecting Phe metabolism. Despite the availability of detection methods and treatments, PKU remains a major global health concern. In addition, genetic variants that may affect the severity and prognosis of PKU have been identified, leading to the need for a more complete and up-to-date understanding of the disease and its clinical implications. The rationale for this literature review is the importance of improving knowledge regarding PKU in childhood, updating screening methods, assessing advances in treatment, and understanding the long-term implications of the disease. Potential limitations were mentioned, such as availability and access to literature, publication bias, geographical and linguistic scope, selection bias, and timeliness of information. The lack of external funding was also a limitation.

Study Conduction

An exploratory documentary study was conducted with the aim of preparing a review of state-of-the-art diagnostic and therapeutic advances in PKU in the pediatric population over the last five years. The search for information was carried out using MESH terms AND and NOT and Boolean operators AND and NOT from search engine repositories and databases, as follows.

PubMed 1st search: ((((((neonatal) AND Infants) AND phenylketonuria) AND Diagnosis) AND Management) NOT Pregnancy) NOT Adult; Filters applied: free full text, review, and 5 years.

PubMed 2nd search: ((((((neonatal) AND Infants) AND phenylketonuria) AND Diagnosis) AND Management) NOT Pregnancy) NOT Adult; Filters applied: free full text, in the last five years.

SCIELO: (phenylketonuria) AND (neonatal) AND (infants) AND (diagnosis) AND (management) AND NOT (adults) AND (pregnancy).

HINARI: (phenylketonuria) AND (neonatal) AND (infants) AND (diagnosis) AND (management) AND NOT (adults) AND (pregnancy) Filters applied: Full Text Online, 5 years, humans, infants, newborn phenylketonuria, medical screening, infant phenylketonuria - diagnosis, diagnosis, neonatal screening, genetics and heredity, newborn screening (NBS), children, and English.

Results

**Figure 1:**
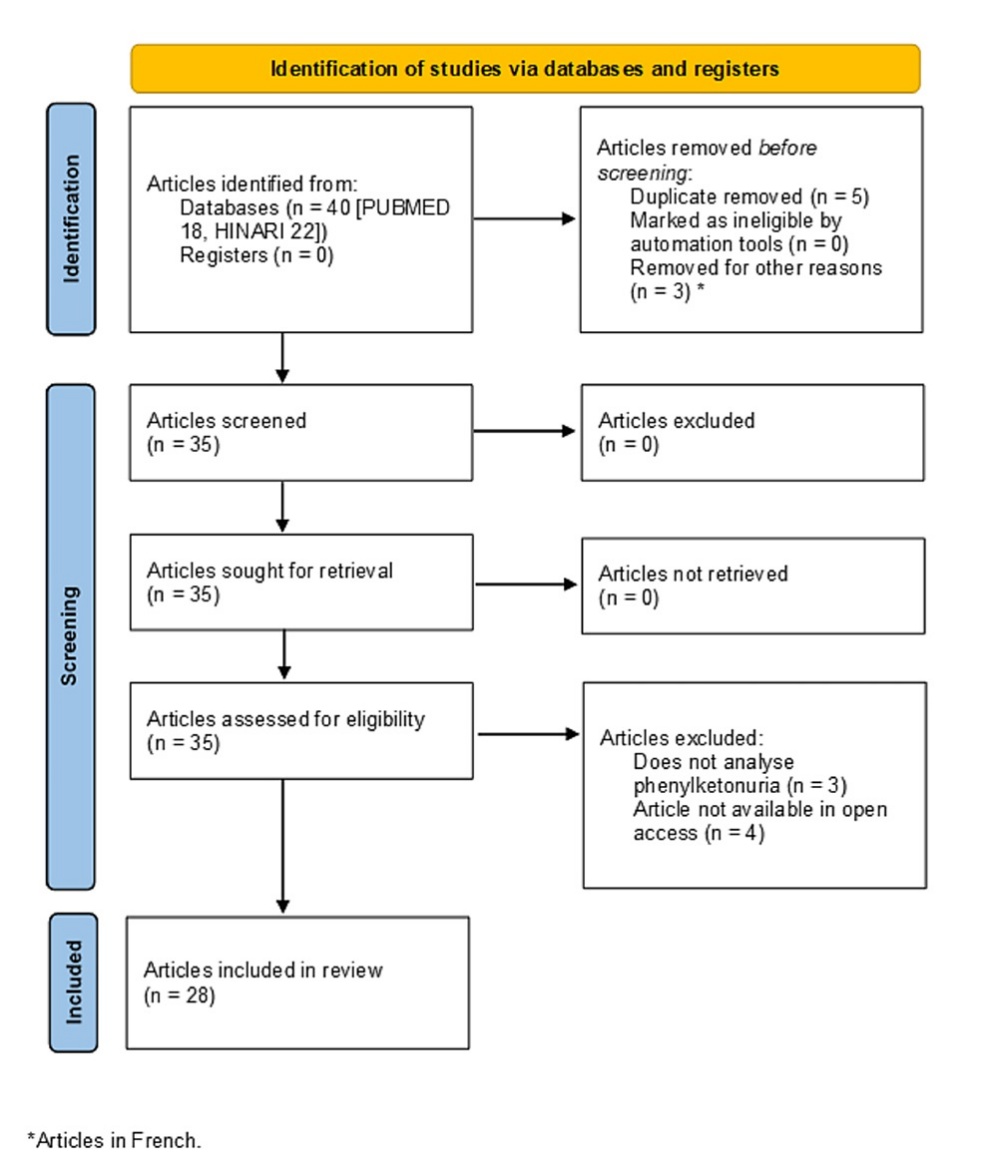
PRISMA 2020 flow diagram It describes the selection flow of manuscripts after their analysis, prior to the elaboration of the discussion.

A total of 40 published articles were obtained as a result of the searches, 22 from HINARI and 18 from PUBMED, in the last five years, from which we proceeded to carry out an analysis of the fulfillment of the inclusion and exclusion criteria. None of the articles were retrieved from the SCIELO database. Figure 1 shows a flowchart used for the selection of articles obtained from different databases or search engines.

Discussion

Diagnosis Innovations

In 1969, in Catalonia, the detection of phenylketonuria was detected in the province of Barcelona in Catalonia; in the rest of Spain, a diagnostic study of neonates with PKU began in 1977, with the approval of the national plan for the prevention of disabilities, using fluorometric methods (the Guthrie test in neonatal screening and the McCamman and Ronis method in neonates exposed to antibiotics) in the first decade of the program, obtaining accreditation of its laboratory in 2005. By 2007, tandem mass spectrometry (MS/MS) technology was incorporated to replace fluorometric tests for the detection of PKU, with a grade of recommendation AI, finalizing the extension of diseases for diagnosis by reference laboratories [15,16].

Support from sectional and national governments in implementing policies for screening inherited metabolic diseases such as PKU is important, as highlighted by Heidari et al. [17].

Heidari et al. [17] emphasized the importance of a multidisciplinary approach in countries such as the UK, Canada, and New Zealand while highlighting the challenges faced in Iran owing to limited technical and human resources. Multidisciplinary management involves the collaboration of various healthcare professionals in implementing screening programs for inherited metabolic disorders such as PKU. In Iran, the lack of capacity in the healthcare system, a shortage of resources, and inadequate laboratory equipment have hindered the national implementation of PKU screening. Overcoming these obstacles and ensuring successful early detection and management of inherited metabolic disorders requires support from the government, including the allocation of resources, strengthening technical capacity, and involving healthcare professionals from different disciplines. The effective implementation of screening policies and timely diagnosis demonstrated in countries such as the UK, Canada, and New Zealand can serve as a model for others to follow.

Haitjema et al. [18] emphasized the importance of effective communication in newborn screening results and its impact on parents' perceptions of information. Poor communication can lead to an increased risk of posttraumatic stress syndrome and hinder acceptance of the child's illness. Transparent and comfortable communication is crucial to ensuring that parents understand the screening results and disease implications of the necessary measures for metabolic control. Effective communication reassures and empowers parents, facilitating their acceptance of the disease and commitment to treatment. Health professionals involved in newborn screening should develop communication skills by providing clear information, addressing concerns, and providing emotional support. It should be emphasized that the study discussed was about parents' perceptions of the communication of results, and that for many, this was 40 years ago.

Newborn screening, introduced in the 1960s in the US, has expanded globally to include tests for PKU. PKU is a metabolic disorder that, if untreated, leads to irreversible mental retardation due to the inability to break down phenylalanine. Newborn screening programs detect PKU early, thereby allowing dietary treatment to prevent mental retardation. Guthrie's classical method played a significant role in establishing PKU screening in the UK. This method uses a bacterial inhibition test to identify elevated phenylalanine levels in the blood shortly after birth. Guthrie also developed a simple system for collecting blood samples on filter paper, facilitating widespread implementation and enabling the detection of other metabolic disorders through the antibacterial testing of other metabolites [19].

A study conducted in Mexico and published in 2021 included 124 PKU patients. It was observed that 78.2% of the patients were diagnosed early by neonatal screening, whereas 21.8% belonged to the clinical diagnosis group. Based on blood phenylalanine concentrations, 50% of the patients had cPKU, 20.2% had moderate PKU (mPKU), and 29.8% had mild hyperphenylalaninemia (MHP). Sixty pathogenic variants in PAH enzyme genotypes were identified, with the most frequent variant being c. 60 + 5G > T. Significant differences were observed in the frequency of six of these variants compared with the data reported in BIOPKUdb. Most pathogenic changes corresponded to missense changes (58%), affected the catalytic domain of the enzyme (56.6%), and involved exon 7. A total of 100 different genotypes were identified, most of which were compound heterozygotes. The most frequent homozygous genotypes were c. [60 + 5G < T], c. [60 + 5G > T], and c. [1162G > A]; [1162G > A]. A 95.2% concordance with the genotypic phenylketonuria (GPV) database was observed. Seventy-four percent of the patients had a genotype identical to that reported in the database, and 86.9% showed phenotypic concordance. The pathogenic variant c 60 + 5G > T was found in 26 patients, and brain damage was observed in two patients homozygous for this variant. This study observed that the general genotype/phenotype concordance was 83.8% when compared with identical genotypes reported in the BIOPKUdb [20].

Vela-Amieva et al. [21] investigated the mutational and phenotypic spectra of PHA deficiency in Zhejiang Province, China. A total of 420 unrelated samples were collected from patients diagnosed with PAH deficiencies between 1999 and 2016. Patients were classified into three phenotypic groups: cPKU, mPKU, and MHP. Patients with BH4 cofactor deficiencies were excluded from the study. Genetic analysis revealed 72 PAH variants, including missense, nonsense, splice site, frameshift, and synonymous mutations. The distribution of variants was uneven across PAH genes, and hotspot regions were identified. Notably, certain variants were specific to particular phenotypic groups. Compound heterozygous alleles were found in 85.8% of patients, whereas monoallelic mutations were observed in the remaining cases. Various genotypic combinations have been identified, and some genotypes are associated with specific phenotypes. The null allele frequency differed significantly between patients with cPKU and mPKU. These findings contribute to our understanding of the genetic basis of PAHD and its phenotypic variability in Zhejiang Province, China.

Öztürk et al. [22] reported that 90.3% of patients were diagnosed during a newborn screening program. 38.7% of the patients attended follow-up appointments with their mothers, 61.3% with both parents, and none with their fathers alone. Mothers responded more accurately than fathers. The mean knowledge scores of mothers and fathers were 73.97 ± 12.72 and 53.04 ± 22.25, respectively. Parents of children older than 13 years of age scored the highest. The study found that the presence of another family member, parents' educational level, employment status, professional qualifications, previous PKU training, and economic status influence parents' knowledge. The importance of providing regular information to patients and their parents to increase their knowledge of the disease and dietary management is highlighted. PKU is an inherited metabolic disorder that requires a Phe-restricted diet and complex management. These results emphasize the need for regular training of patients and their parents to ensure compliance with dietary treatment and control food intake. In addition, the importance of active parental involvement in the management of PKU, especially during infancy, is highlighted as it plays a key role in dietary adaptation and nutrient selection.

Öztürk et al. [23], in their study, described an increase in false-positive cases in the detection of hyperphenylalaninemia (HPA) in newborns due to the contamination of blood sample collection devices during the printing process. Contaminants were identified on the collection devices, which generated positive results in fluorometric tests but negative results in pooled mass spectrometry (MS/MS). The contaminants were extracted with 80% ethanol and showed an absorption peak at approximately 245 nm, suggesting that they might contain benzene derivatives with a structure similar to that of Phe. High-performance liquid chromatography (HPLC) analysis identified prominent peaks specific to contaminated devices, including methyl-2-benzoylbenzoate (MBB CAS#606-28-0). This report highlights the importance of regulating and controlling the quality of sample collection devices to avoid contamination and false-positive results in HPA detection.

Foreman et al. [24] examined the global prevalence of PAH deficiency, an autosomal recessive disease that causes elevated blood Phe concentrations. They conducted a systematic literature review and meta-analysis to estimate the worldwide prevalence of PAH from newborn screening studies, as well as regional differences and Phe cut-off values used in confirmatory testing. After evaluating 85 publications, the data from 45 publications that met the quality criteria were included in the meta-analysis. The overall prevalence of PAH was estimated at 0.64 per 10,000 births, with significant regional variation. The Phe cut-off values used in the confirmatory tests also showed differences in prevalence. These results highlight the regional variability in PAH prevalence and the need for a personalized approach to disease detection and management. However, they highlighted the limitations of the relatively small sample sizes of the available studies.

In a six-year retrospective study conducted in central Saudi Arabia, 56,632 newborns were screened for disorders using NBS tests. Seventeen disorders were assessed using blood samples collected 24 hours after birth. A second sample was collected to obtain an initial positive result. During the study period, 38 cases of disorders detected by NBS were confirmed, representing an incidence rate of congenital hypothyroidism of 1 in 3,775 newborns. Propionic aciduria was the most common metabolic disorder, with an incidence of one in 14,158 patients. Very long-chain acyl CoA dehydrogenase deficiency and glutaric aciduria type 1 had incidences of 1 in 18,877 patients. PKU, biotinidase deficiency, maple syrup urine disease (MSUD), and citrullinemia had incidences of 1 in 28,316 patients each. However, galactosemia and 3-methylcrotonyl carboxylase deficiency had the lowest incidence, with one case per 56,632 newborns [25].

Mohamed et al. [26] in their study visualized a decision analysis model to estimate and compare the long-term costs (80 years) and health effects of neonatal screening for PKU and congenital hypothyroidism. The data were obtained from the literature and adjusted for Swedish conditions. A societal perspective was adopted, considering the costs to healthcare providers, municipal services, and lost productivity due to morbidity. The results showed that screening for PKU in 100,000 newborns resulted in 73 quality-adjusted life years (QALYs) compared to no screening. When congenital hypothyroidism was added, the number of QALYs gained was 232 compared to PKU screening alone, for a total of 305 QALYs gained. The corresponding costs were $80.8, $70.3, and $10.05 million for no screening, PKU screening, and PKU plus congenital hypothyroidism screening, respectively. This indicates that PKU plus congenital hypothyroidism screening is more effective and less costly than other strategies. Most of the cost savings with PKU plus congenital hypothyroidism screening were due to reductions in productivity losses and municipal service costs. In conclusion, the Swedish neonatal screening program for PKU and congenital hypothyroidism saves substantial costs to society while generating additional QALYs, emphasizing the importance of public investment in early diagnosis and treatment.

Appelberg et al. [27] prepared external standards to quantify phenylalanine in dried blood samples. Blood was obtained in heparin form from a healthy donor, adjusted for hematocrit to 50%, and divided into six portions. Each portion was spiked with different concentrations of phenylalanine to create standards in the range of 0-800 μmol/l. The samples were placed on filter paper and allowed to dry. Phenylalanine elution was then performed using methanol and analyzed by LC-MS/MS. The method was validated through linearity, precision, accuracy, and stability tests. The results obtained showed a good correlation with other established methods, such as ELISA and mass spectrometry. The developed method proved to be accurate and reliable for the quantification of phenylalanine in dried blood samples, making it suitable for PKU diagnosis and monitoring.

After testing positive for PKU in neonatal screening by tandem mass spectrometry, an elevated phenylalanine concentration (>120 μmol/L) and increased phenylalanine/tyrosine ratio (>3 if tandem mass spectrometry is used) should be confirmed in a second blood sample. All causes of neonatal phenylketonuria should be ruled out, including liver disease, PAH deficiency, and genetic defects in the synthesis or regeneration of BH4 or DNAJC12. In addition, further investigations and genotyping should be performed in the neonatal period to assess the likelihood of a response to BH4 and guide treatment options. To perform BH4 load testing in newborns, a baseline capillary blood sample was collected to measure phenylalanine levels, followed by the administration of sapropterin (20 mg/kg) dissolved in breast milk or infant formula. Capillary blood samples were collected to measure phenylalanine levels 4, 6, 8, 12, 16, and 24 h after sapropterin administration. The results should be available within 24 hours of the end of the test, and treatment can then be implemented. The interpretation of the BH4 loading test results was based on a decrease in the blood phenylalanine concentration. A decrease of ≥30% at any time during the test indicated the presence of a BH4-sensitive HPA. A rapid decrease in Phe concentration towards the target range suggests a genetic disorder in BH4 synthesis. If the Phe concentration decreases to the target range within 24 hours, this suggests a DHPR deficiency or a fully BH4-sensitive PAH deficiency. These results should be critically reviewed and confirmed in long-term treatment trials to determine a significant benefit in terms of Phe tolerance. Patients with a Phe decrease greater than 30% may undergo a one-month trial with sapropterin alone if Phe levels fall within the target range, or combined with a Phe-restricted diet if PHe levels remain above the target range [28].

Muntau et al. [29] analyzed the effects of breast milk sucking (EBMS) on alleviating pain during neonatal heel blood sampling. A randomized controlled trial was conducted involving 96 neonates who were divided into two groups. The observation group received the EBMS intervention, whereas the control group received the routine intervention. The results showed that the observation group had a higher success rate and a shorter blood collection time. Heel congestion also improved, and crying and bleeding times were reduced compared with those in the control group. Additionally, the observation group had higher blood oxygen saturation, a lower heart rate, and lower Neonatal Infant Pain Scale (NIPS) scores. The EBMS can enhance the efficiency of blood collection, maintain normal physiological parameters, and effectively reduce pain and complications associated with neonatal heel blood sampling.

Wu et al. [30] described the development and validation of a multiplex SNaPshot minisequencing method to detect common mutations in the PAH gene in Iranian patients with PKU. The multiplex SNaPshot assay allows for the simultaneous analysis of multiple single nucleotide polymorphisms (SNPs) in a single reaction. The researchers selected ten common pathogenic mutations based on previous studies conducted in different regions of Iran. Specific primers were designed for the amplification and minisequencing of the target regions. The assay demonstrated 100% sensitivity and specificity for the detection of selected mutations. This method can be used as a quick and affordable confirmation test for the early genotyping of patients with PKU following positive neonatal screening results. Sanger sequencing was performed to validate the multiplex minisequencing assay results. Overall, this study presents a reliable and efficient method for detecting common PAH gene mutations in Iranian patients [30].

Namdar Aligoodarzi et al. [31] reviewed the metabolic data of 304 patients with PAH deficiencies detected during newborn screening over the past 37 years. Initial management consisted of Phe elimination through the exclusive administration of a Phe-free formula until Phe normalization. Based on the genotype and Phe tolerance assessed during follow-up, 55 patients had cPKU (18%), 50 had mild PKU (17%), and 199 had non-PKU hyperphenylalaninemia (65%). After Phe elimination, phenylalanine was gradually reintroduced into the diet to assess individual metabolic phenotypes. Phe tolerance was stable in classical PKU (~200 mg/day) but increased in milder forms, allowing an unrestricted diet in non-PKU hyperphenylalaninemia. Phe washout in infants with PKU ensures early correction of hyperphenylalaninemia and facilitates the rapid definition of individual tolerance, allowing optimal dietary personalization and longitudinal metabolic control.

Porta et al. [32] reviewed the metabolic data of 304 patients with PAH deficiency detected during newborn screening over the past 37 years. The initial management consisted of phenylalanine elimination through exclusive administration of a Phe-free formula until the normalization of phenylalanine. Based on the genotype and phenylalanine tolerance assessed during follow-up, 55 patients had classical PKU (18%), 50 had mild PKU (17%), and 199 had non-PKU hyperphenylalaninemia (65%). After Phe elimination, phenylalanine was gradually reintroduced into the diet to assess individual metabolic phenotypes. Phe tolerance was stable in classical PKU (~200 mg/day) but increased in milder forms, allowing an unrestricted diet in non-PKU hyperphenylalaninemia. Phe washout in infants with PKU ensures early correction of hyperphenylalaninemia and facilitates the rapid definition of individual tolerance, allowing optimal dietary personalization and longitudinal metabolic control.

A case report of a 4-day-old male patient who presented with hyperphenylalaninemia detected during newborn screening was studied. His family history indicated consanguineous parents and a deceased brother with 6-pyruvoyltetrahydropterin synthase (PTPS) deficiency. Initial investigations revealed persistent hyperphenylalaninemia with low biopterin levels, suggesting a PTPS deficiency. However, genetic sequencing revealed that the patient was heterozygous for a familial PTPS variant. Further analysis revealed a homozygous pathogenic variant in the PAH gene, explaining hyperphenylalaninemia. Interestingly, the parents were carriers of two diseases that cause hyperphenylalaninemia. This case highlights the challenges of differentiating between homozygotes and heterozygotes in metabolic disorders and the potential impact of multiple variants in genes along a metabolic pathway on clinical and biochemical outcomes [33].

This study evaluated the effectiveness of post-analytical tools in triaging newborn screening (NBS) results and reducing the need for confirmatory testing. The researchers extracted data from a follow-up database in Georgia, focusing on positive screens for four conditions: PKU, MSUD, MCAD deficiency, and VLCAD deficiency. They analyzed the outcomes of the initial NBS, the use of post-analytical tools, and the need for confirmatory testing. The results showed that the post-analytical tools improved the positive predictive value (PPV) for PKU screening, whereas the PPV for MCAD and VLCAD deficiency remained lower. Implementing these tools earlier in the screening process could reduce false-positive results and improve the efficiency of the NBS follow-up system [34].

Hall et al. [35] evaluated the incidence and genetic characteristics of HPA in Xiamen, China. Data from neonatal HPA screenings obtained using a fluorometric method and tandem mass spectrometry (MS/MS) from 2013 to 2017 were analyzed. Suspected positive samples were subsequently diagnosed using MassArray, multiplex ligation-dependent probe amplification (MLPA), and Sanger sequencing. A total of 418,831 newborns were screened, of whom 19 were diagnosed with APH, with an incidence of 1:22,044. Of these HPA patients, 15 were positive for PKU (1:27,922), and four were positive for tetrahydrobiopterin deficiency (BH4D, 1:104,708). A total of 17 mutations among the 38 alleles were identified in 19 patients, with a detection rate of 94.74%, including 13 mutations in PAH and four mutations in PTS. Among these, c.721C>T, c.728G>A, c.1197A>T, c.611A>G, and c.331C>T, and c.259C>T and c.155A>G mutations were the most prevalent PAH and PTS mutations, respectively, in Xiamen. Therefore, this study systematically demonstrated the incidence and spectrum of PAH mutations in Xiamen. This information could contribute to genetic counselling, prenatal diagnosis, and the management of patients with PAH. Furthermore, the combination of MS/MS technology with molecular genetic diagnosis is an effective strategy for the future neonatal screening of PAH in Xiamen.

Wang et al. [36], conducted at Sohag University Hospital, Egypt, focused on selective screening for inborn errors of metabolism (IEM) using tandem mass spectrometry. In total, 308 participants with suspected IEM were recruited and underwent metabolic screening. Among the neonates screened, 30.2% (93) were diagnosed with IEM. Phenylketonuria was the most common diagnosis, followed by glutaric aciduria type 1, and maple syrup urine disease (43%, 19.4%, and 14%, respectively). Other metabolic disorders were identified in a smaller number of patients. Most patients showed significant improvement with appropriate management. In conclusion, neonatal screening for IEM using tandem mass spectrometry is recommended for the early diagnosis and management of this group of disorders.

The impact of the COVID-19 pandemic on the diagnosis and management of IEM has been significant globally. The pandemic has affected healthcare systems worldwide, leading to disruptions in services and a decrease in the number of patients. The lack of access to specialized laboratories and the need for multidisciplinary medical teams have hampered the diagnostic procedures and treatment of patients with IEM. In addition, patients with IEM are at an increased risk of serious complications if infected with SARS-CoV-2 because of their pre-existing chronic conditions and vulnerable immune systems. Most IEM centers have experienced a significant decrease in patient visits, laboratory specimens, and new diagnoses during the pandemic. Measures, such as online medical consultations, home delivery of formulas and medicines, and home delivery of dried blood samples, have been implemented to mitigate these negative effects. It is important to establish coordinated response strategies between the medical community and healthcare policymakers to address future pandemics and ensure adequate care for patients with IEM [37].

The impact of the COVID-19 pandemic on the diagnosis and management of IEM has been significant globally. The pandemic has affected healthcare systems worldwide, leading to disruptions in services and a decrease in the number of patients. The lack of access to specialized laboratories and the need for multidisciplinary medical teams have hampered the diagnostic procedures and treatment of patients with IEM. In addition, patients with IEM are at an increased risk of serious complications if infected with SARS-CoV-2 because of their pre-existing chronic conditions and vulnerable immune systems. Most IEM centers have experienced a significant decrease in patient visits, laboratory specimens, and new diagnoses during the pandemic. Measures, such as online medical consultations, home delivery of formulas and medicines, and home delivery of dried blood samples, have been implemented to mitigate these negative effects. It is important to establish coordinated response strategies between the medical community and healthcare policymakers to address future pandemics and ensure adequate care for patients with IEM [38].

Therapeutic Innovations

Treatment of PKU should begin as soon as possible after detection, preferably in the first week of life. Depending on Phe levels in the blood, it is necessary to exclude this amino acid from the diet or provide a Phe-restricted diet. In many cases, breast milk is used in combination with therapeutic formulas. Early initiation of treatment requires the implementation of a rapid neonatal screening programme and adequate follow-up and follow-up testing materials [20]. Sometimes, primary care providers can communicate openly with the baby's family to access the necessary specialized care. Generally, PKU management involves a drastic reduction in dietary protein sources to reduce Phe intake. Because Phe is an indispensable amino acid, total body growth cannot occur if it is completely eliminated from the diet. Individual "tolerance" to phenylalanine may vary among children.

Al Bari [20] stressed the importance of initiating PKU treatment early to minimize the harmful effects of Phe accumulation in the body. The need to exclude this amino acid from the diet or follow a phenylalanine-restricted diet was mentioned. In addition, the importance of open communication between primary care providers and the baby's family is emphasized to ensure access to necessary specialized care. The importance of reducing dietary protein intake has been mentioned, but it has also been highlighted that phenylalanine is an essential amino acid and that tolerance to phenylalanine may vary from individual to individual.

Al Bari et al. [20] discussed various aspects related to the treatment of PKU. They highlighted the importance of assessing the necessary amount of Phe in each child's diet and how this is achieved by consuming measured amounts of specialized PKU formula, which contains all the amino acids necessary for growth except Phe. In the first few days of life, breastfeeding or regular formula intake may be temporarily suspended to allow a rapid decrease in Phe levels. During this period, the infant may consume only Phe-free PKU formula. Once levels begin to decrease, breastfeeding or regular formula can be reintroduced along with PKU formula until Phe levels remain within the target range. Different therapeutic approaches for the development of PKU have been described, such as the use of cofactors such as BH4 and improvements in dietary palatability with functional foods such as glycomacropeptide (GMP) and large neutral amino acid (LNAA) supplementation. However, long-term toxicological studies are required to assess the full impact of these treatments. In addition, advances in gene therapy and enzyme replacement therapy targeting the liver, such as the use of phenylalanine ammonium lyase (PAL) and the application of PAL conjugated to PEG, have been reported. This highlights the importance of identifying safe and effective alternative therapies for all patients with PKU, regardless of genotype, phenotype, or sex differences.

Treatment of PKU involves reducing protein sources in the diet to reduce Phe intake, and nutritionists play an essential role in helping families manage their diet. Phe levels can be monitored using blood tests obtained from the heels or fingertips. The role of family physicians in establishing consistent medical care, counselling, and family support is critical. In addition, it is important to educate mothers about PKU and their adherence to diet, as this is crucial for the care and maintenance of patients with PKU. Patients with PKU are susceptible to carbohydrate intolerance and insulin resistance, especially older and obese patients. This also highlights the importance of being aware of dietary supplements that may contain aspartame, as this can increase Phe levels and the economic burden of treating PKU, especially the costs of therapeutic foods and supplements. Therefore, it is necessary to implement government subsidies to cover these costs and provide social support to patients [20].

The treatment of newborns with BH4-sensitive PAH deficiency using sapropterin can follow two scenarios: if the results of the BH4 loading test were obtained on the day after the test, treatment with BH4 was started immediately (in the case of a complete response); if the results cannot be obtained within this time frame, dietary treatment should be started, either with phenylalanine-free medical foods if the baseline Phe value is >600 μmol/L or with a mixed breast milk/full-infant formula diet if the baseline Phe value is <600 μmol/L, until test results are obtained. In these cases, there is a high probability of sensitivity to BH4. If the results indicate a BH4-sensitive PAH deficiency, pharmacotherapy with sapropterin can be initiated. Sapropterin can be administered to infants with complete responses. Although some clinicians prefer a starting dose of 20 mg/kg, the recommended starting dose is 10 mg/kg/day. A single dose of sapropterin, dissolved in milk, was administered in the morning. Blood Phe concentrations should be closely monitored (preferably daily), and Phe intake should be adjusted gradually to remain within the target range. The algorithm for adjusting Phe intake was based on the average blood Phe concentration. After several weeks, the maximum Phe intake that could be tolerated while maintaining blood Phe concentrations within the target range could be determined. A critical assessment of the benefits of sapropterin treatment was made by considering the patient's response in terms of phenylalanine tolerance. For children under four years of age who are already receiving dietary treatment, additional BH4 sensitivity testing may be performed, and a trial of sapropterin treatment is recommended if the BH4 loading test is not possible [29].

In a study of 95 European centres, dietary practises for the treatment of PKU were analyzed. The results showed that more than 50% of the centres performed neonatal screening in the first three days of life. At the time of diagnosis, 61% of the centres were hospitalized. Initiation of a low-phenylalanine (Phe) diet occurred before 10 days of age in >60% of the centres. Although 42% of the infants were breastfed at diagnosis, this figure decreased to 26% later in life. The average duration of breastfeeding was 4-26 weeks. In addition, geographical differences have been observed in dietary practises and Phe levels [39].

Berry et al. investigated the growth, protein, and energy intake of children with PKU who consumed a weaning protein substitute during the first two years of life. This case-control study included 20 children with PKU and 20 healthy controls (HCs). The results showed that both groups had normal growth parameters. Children with PKU had consistent protein intake from protein substitutes, whereas the controls had a wider range of protein intake. Carbohydrate intake was higher in the PKU group from one to two years of age, whereas fat intake was higher in the control group after 12 months. Both groups exceeded the recommended safe protein intake level. Overall, this study suggests that weaning protein substitutes effectively support protein and energy intake in children with PKU. There was no significant difference in the total energy intake or percentage of energy intake from food between the PKU and control groups. The total energy intake and energy intake as a percentage of the dietary reference values (DRV) were similar longitudinally and at all ages. No sex differences were observed within or between the groups. The percentage of energy from protein remained consistent at approximately 14-15% for children with PKU, while it gradually increased from 8% to 17% in control children. Carbohydrate intake as a percentage of energy increased steadily in the PKU group from 49% to 60%, whereas the control group had a more variable and lower carbohydrate intake, ranging from 47% to 54%. Fat intake as a percentage of energy was higher in the control group (34-38%) than in the PKU group, where it decreased from 40% to 25% as carbohydrate intake increased [40].

Medical foods are specially formulated products designed for individuals with a limited capacity to metabolize ordinary foods due to specific diseases or conditions. They play a crucial role in managing IEMs and have been successful in preventing disability and death. The use of medical foods was initially classified as a drug but was later redefined as a distinct category called medical foods to facilitate innovation and accessibility. However, despite their proven efficacy and endorsement by medical organizations, access to medical foods in the United States is not ensured because many health insurance plans do not sufficiently cover them. This lack of coverage poses significant challenges for individuals with IEMs, leading to adverse health outcomes and increased healthcare costs. There is a need for improved access to and reimbursement for medical foods and for potential solutions to address these issues [41].

Over a period of almost 20 years, neonatal screening for hyperphenylalaninaemia (HPA) was performed on 1,292,622 newborns in China. A total of 181 HPA cases were diagnosed, with an incidence rate of 1 in 6,873 patients. All patients were treated before the age of one month. It was found that 97.79% of the patients had PAH deficiency and 2.21% had BH4D deficiency. Significant differences in Phe concentrations were observed among different PAHD types. Forty mutations have been detected in patients with PAHD, the most common being the c.728G > A (p.Arg243Gln) mutation. Relationships between PAHD genotypes and biochemical phenotypes have been established [33].

Yuskiv et al. [42] surveyed dietitians in Canada to understand their current practises in the nutritional management of paediatric patients with PAH deficiency. The survey covered various aspects of PAH deficiency management, including disease severity classification, monitoring of biomarkers, recommended dietary intake, use of medical foods and supplements, clinic visits, communication with patients and families, and adherence to therapy. The results showed that dietitians were aware of the published guidelines and used a combination of pretreatment blood phenylalanine levels, Phe tolerance, and PAH genotypes to classify disease severity. Most dietitians initiated dietary treatment at blood Phe levels ≥360 μmol/L and monitored the Phe and tyrosine levels. Clinical visits were most frequent among infants and declined with age. Dietitians have used various methods to assess patients' adherence to the diet and employed strategies, such as individualized nutrition counselling, to improve adherence. Overall, this study provides insights into the current practises of dietitians in Canada regarding the nutritional management of PAH deficiency in paediatric patients.

Walkowiak et al. [8] analyzed treatment adherence in children under five years of age with PKU. A retrospective longitudinal study was conducted on patients diagnosed with classical PKU between May 1999 and September 2010 at the Department of Paediatric Gastroenterology and Metabolic Diseases, Poznan University of Medical Sciences, Poland. Patients who required a low-phenylalanine diet to maintain plasma Phe levels within the target range of 2-6 mg% (120-360 μmol/L) were included. Records of each patient's visit to the specialist were collected and compared with recommendations for the frequency of visits. Blood Phe concentrations during the first five years of life were also assessed, and the dietary control index (DCI) and the percentage of Phe concentrations within the therapeutic range were calculated. During the first five years of life, children with PKU visited the specialist an average of 14.1 times, and 7,638 blood tests were performed to measure Phe levels. The number of visits and blood tests performed has decreased over the years. During the first year, the average number of visits was 4.7, whereas in the fifth year, it was 1.7. Compliance with visit recommendations was the lowest in the third year (62%). Decreases in blood Phe levels have been observed over the years. The study found that in the first year of life, 56% of patients had less than 20% Phe test results within the therapeutic range, whereas in the fifth year, only 26% had out-of-range results. In addition, significant correlations were found between the percentage of Phe concentrations within the therapeutic range, the number of specialist visits, and the frequency of blood Phe monitoring. No significant correlations were found between parental education level and family socioeconomic status, the percentage of Phe concentrations within the therapeutic range, and the IDC. The study concluded that there is variability in treatment adherence in children with PKU under five years of age and highlighted the importance of regular follow-up and adequate monitoring of blood Phe levels to maintain optimal dietary control.

Key Points

Methods for the diagnosis and treatment of PKU in childhood: This text highlights the importance of neonatal screening programmes for the early detection of PKU. Various diagnostic methods, such as fluorometric tests and MS/MS technology, have been used to detect PKU. Genetic analysis has identified different genotypes and variants of Phe hydroxylase that are associated with PKU. Furthermore, communication between healthcare professionals and parents plays a crucial role in conveying screening results and ensuring effective disease management. The treatment involved excluding Phe from the diet or providing a Phe-restricted diet. Specialized PKU formulas and breastfeeding are often used in combination. The text also mentions the potential use of cofactors, such as BH4, and advancements in gene therapy and enzyme replacement therapy targeting the liver.

Innovations in the early diagnosis of PKU: This study highlights the significance of neonatal screening in the first days of life for the early diagnosis of PKU. Geographical differences exist in the timing of dietary treatment initiation. There is also ongoing research on BH4 sensitivity testing and the use of sapropterin in newborns with BH4-sensitive PAH deficiency. The development of genetic testing methods and the identification of specific mutations associated with PKU are potential innovations. The implementation of neonatal screening programmes has led to the early diagnosis of PKU. The classical method developed by Guthrie, which uses bacterial inhibition tests and blood samples collected on filter paper, plays a significant role in PKU screening. However, it is important to address issues such as contamination of blood sample collection devices to avoid false-positive results.

New strategies for the treatment and management of PKU: Dietary management of PKU involving a Phe-restricted diet is crucial for preventing mental retardation. This study highlights the need for regular training for patients and their parents to ensure compliance with dietary treatment and control food intake. Active parental involvement, particularly during infancy, is essential to dietary adaptation and nutrient selection. Advancements in gene and targeted therapies have offered new strategies for the treatment of PKU. This review discusses several strategies for the treatment and management of PKU. These include reducing dietary protein sources, individualizing Phe intake, and monitoring Phe levels through blood tests. Dietitians play a crucial role in helping families manage their diets. The importance of adherence to the diet and awareness of dietary supplements containing aspartame is emphasized. The economic burden of PKU treatment and the need for government subsidies were also mentioned.

Advances in gene therapy and specific targeted therapies for PKU: Although the texts provide limited information on gene therapy and specific targeted therapies for PKU, they mention the identification of genotypes and variants associated with PKU. This suggests the need for ongoing research and exploration of genetic factors to develop new treatment approaches. The text reports advances in gene therapy, specifically targeted therapies for PKU. These include the use of PAL and PAL conjugated to polyethylene glycol (PEG). The potential of gene and enzyme replacement therapies that target the liver has been highlighted. Further long-term toxicological studies are required to assess the safety and efficacy of these treatments.

Key findings and recommendations for clinical practise: These findings emphasize the importance of multidisciplinary approaches, government support, and resource allocation for the successful implementation of PKU screening programmes. Effective communication with parents, the provision of clear information, and emotional support are crucial for ensuring understanding and acceptance of the disease. The texts also highlight the regional variability in the prevalence of PKU and the need for personalized approaches to detection and management. Additionally, cost-effectiveness analyses support the benefits of neonatal screening for PKU and congenital hypothyroidism. Key findings from the texts include the importance of early treatment initiation, regular follow-up, and Phe level monitoring. The significance of dietitians in providing nutritional management and counselling has been emphasized. Adherence to a diet and awareness of dietary supplements are crucial. Access to medical foods and reimbursement for PKU treatment are challenges that require potential solutions and government subsidies. This text underscores the need for ongoing research and advancements in diagnostic methods, treatment strategies, and alternative therapies for all PKU patients.

## Conclusions

The reviewed texts provide valuable information on PKU and highlight the importance of early detection and prompt treatment to minimize the harmful effects of Phe accumulation in the body. They highlighted the need for effective communication between primary care providers, specialists, and families to ensure adequate access to necessary care. In addition, different methods of diagnosis and treatment have been presented, including the exclusion of Phe from the diet and the use of specialized formulas and foods. PKU management is based on personalized dietary approaches, regular monitoring of Phe levels, and the education of mothers and families. In addition, continued research, government subsidies, and social support are important to improve access to medical foods and cover the costs associated with treatment.

Recommendations derived from these texts include the implementation and promotion of neonatal screening programmes for PKU as well as the immediate initiation of treatment once a diagnosis is made. Effective communication and collaboration between health professionals and families are essential to ensuring timely access to necessary care. In addition, the use of improved diagnostic methods such as BH4 sensitivity testing is suggested, and the importance of following a low-Phe diet or eliminating Phe altogether is emphasized. Patients and their families should be provided with information on specialized formulas and foods available to maintain adequate levels of Phe in the diet. In terms of management, it is recommended that nutritionists or dietitians be involved in the development and management of personalized dietary plans. Regular monitoring of Phe levels is essential to ensure dietary compliance and make necessary adjustments to treatment. Education and support should also be provided to mothers and families regarding the importance of strict dietary adherence and proper management of PKU. In addition, research on additional therapies, such as cofactors and targeted treatments, is encouraged to evaluate their efficacy and potential benefits in the treatment of PKU. Ongoing research is needed to improve diagnostic techniques, treatment options, and outcomes for people affected by PKU. Finally, the importance of financial support and access to care was highlighted. It is essential to advocate for government grants and social support to improve access to medical food and cover the costs associated with PKU treatment. Awareness should be raised regarding the economic burden of the disease and the need for financial support to ensure optimal care for all patients. In addition, it is important to keep up with the pace of development.
